# A data-driven artificial intelligence model for remote triage in the prehospital environment

**DOI:** 10.1371/journal.pone.0206006

**Published:** 2018-10-23

**Authors:** Dohyun Kim, Sungmin You, Soonwon So, Jongshill Lee, Sunhyun Yook, Dong Pyo Jang, In Young Kim, Eunkyoung Park, Kyeongwon Cho, Won Chul Cha, Dong Wook Shin, Baek Hwan Cho, Hoon-Ki Park

**Affiliations:** 1 Convergence Research Center for Diagnosis, Treatment, and Care of Dementia, Korea Institute of Science and Technology, Seoul, South Korea; 2 Department of Biomedical Engineering, Hanyang University, Seoul, South Korea; 3 Smart Healthcare & Device Research Center, Samsung Medical Center, Sungkyunkwan University School of Medicine, Seoul, South Korea; 4 Department of Emergency Medicine, Samsung Medical Center, Sungkyunkwan University School of Medicine, Seoul, South Korea; 5 Department of Digital Health, Samsung Advanced Institute for Health Sciences & Technology, Sungkyunkwan University, Seoul, South Korea; 6 Department of Family Medicine, Samsung Medical Center, Sungkyunkwan University School of Medicine, Seoul, South Korea; 7 Department of Medical Device Management and Research, Samsung Advanced Institute for Health Sciences & Technology, Sungkyunkwan University, Seoul, South Korea; 8 Department of Family Medicine, Hanyang University College of Medicine, Seoul, South Korea; University Technology Petronas, MALAYSIA

## Abstract

In a mass casualty incident, the factors that determine the survival rate of injured patients are diverse, but one of the key factors is the time for triage. Additionally, the main factor that determines the time of triage is the number of medical personnel. However, when relying on a small number of medical personnel, the ability to increase survivability is limited. Therefore, developing a classification model for survival prediction that can quickly and precisely triage via wearable devices without medical personnel is important. In this study, we designed a consciousness index to substitute the factor by manpower and improved the classification accuracy by applying a machine learning algorithm. First, logistic regression analysis using vital signs and a consciousness index capable of remote monitoring through wearable devices confirmed the high efficiency of the consciousness index. We then developed a classification model with high accuracy which corresponds to existing injury severity scoring systems through the machine learning algorithms. We extracted 460,865 cases which met our criteria for developing the survival prediction from the national sample project in the national trauma databank which contains 408,316 cases of blunt injury and 52,549 cases of penetrating injury. Among the dataset, 17,918 (3.9%) cases died while the other survived. The AUCs with 95% confidence intervals (CIs) for the different models with the proposed simplified consciousness score as follows: RTS (as baseline), 0.78 (95% CI = 0.775 to 0.785); logistic regression, 0.87 (95% CI = 0.862 to 0.870); random forest, 0.87 (95% CI = 0.862 to 0.872); deep neural network, 0.89 (95% CI = 0.882 to 0.890). As a result, we confirmed the possibility of remote triage using a wearable device. It is expected that the time required for triage can be effectively reduced by using the developed classification model of survival prediction.

## Introduction

Triage is a method used to prioritize clinical options according to the severity of injured patients who need first aid [1], thereby-aiming to improve the survival rate by providing effective treatments. Among the various types of triage methods, the most basic form is the way in which on-site practitioners evaluate many trauma patients and identify treatment priorities during a mass casualty incident (MCI), such as a natural disaster, fire, terrorism, battlefield, etc. [1]. Although triage based on the diagnosis of clinicians is the most reliable method, it can take a long time for a small number of specialists to directly determine the conditions of many injured patients. For example, based on a review of 4,596 casualties on the battlefield from 2001 to 2011, 87.3% of all injury- related deaths occurred before triage, and 24.3% of these injuries were estimated to be potentially survivable [2]. In such an MCI situation, it is crucial to quickly identify the states of all trauma patients and determine the priority of treatment to increase the survival rate However, the most obvious solution to minimize the time spent on triage is employing enough medical personnel; a shortage of medical manpower results in a decrease in the survival rate of the wounded.

Therefore, it is necessary to develop algorithms that do not rely on medical personnel that can accurately classify trauma patients in a limited time.

Studies quantifying the conditions of injured patient, to classify the severity, began around the 1970s. A representative method is the abbreviated injury scale (AIS) method, which was first designed in 1969 [3] and reported in 1971 [4]. Subsequently, the injury severity score (ISS) [5], which scored the severity according to the complexity of an injury, was developed based on AIS. Thus, AIS and ISS were used in the fields of emergency medicine and critical care medicine in the 1970s; based on the professional experience and judgment of clinicians, these were the most reliable trauma severity scoring systems. Since the 1980s, more studies have investigated the scoring and classification of traumatic injuries based on biological information [6–9]. These methods scored the severity of injuries and estimated survival probabilities according to severity scores using statistical methods. These scoring systems focused on classifying the acute scale of injured patients in a short period of time; one such method relies on the revised trauma score (RTS). A revised version of the trauma score (TS), reported in 1981, RTS classifies the acute scale and survival probability using three indicators: the systolic blood pressure (SBP), respiration rate (RR), and Glasgow Coma Scale (GCS) [7]. The trauma and injury severity score (TRISS) was also developed by utilizing existing scoring systems. As an upgraded version of RTS, TRISS is scored by adding the age and ISS score of an injured patient to the SBP, RR, and GCS values used for RTS scoring [8,9]. In addition, TRISS has been applied to different scoring forms according to the type of trauma, such as blunt or penetrating.

RTS and TRISS were developed to improve the efficiency and accuracy by simplifying previous methods; however, they have not ruled out all medical judgment indicators (e.g., ISS and GCS). Because GCS is a scoring system that summarizes three scaled values (i.e., eye opening, verbal response, and motor response) to classify a patient’s conscious state from a value of 3 to 15 [10], the weight of GCS is higher than those of SBP and RR in the regression equation for RTS scoring [7]. These results demonstrate that medical judgment indicators are still more influential on the classification results than vital signs during the triage of trauma patients. Similar to TRISS, ISS and GCS are also used as important indices to quantify the severity of trauma; however, both indicators have certain limitations in applications where medical personnel cannot directly identify injured patients. Therefore, the main objective of this study is to develop a casualty classification model with high accuracy based on minimum vital signs while replacing medical judgment indicators. For this study, we formed two hypotheses. First, as an indicator for triage, GCS can be replaced with a simplified consciousness score. In previously reported studies that scored consciousness, it has already been shown that GCS can be simplified for certain situations [11,12]. Thus, we hypothesized that GCS can be substituted with a simplified index when it is applied to the casualty classification algorithm for triage. Second, machine learning techniques can improve the accuracy of the casualty classification model. Machine learning approaches help a computer learn all of the complicated and non-linear interactions between variables by optimizing the error between predicted and observed outcomes [13]. Machine learning methods have shown improved prediction performance in many medical and clinical applications [14]. Based on these advantages, recent studies have adopted machine learning to develop clinical decision support models [15–18].

In this study, we developed a casualty classification model based on machine learning approaches for triage in mass casualty incidents by using a simplified consciousness score and vital signs that can be remotely monitored through wearable devices, without relying on medical practitioners.

## Materials and methods

In order to develop proposed casualty classification model, we used four types of machine learning algorithms; logistic regression [19], random forest [20], neural network [21], which were trained and evaluated with the National Trauma Data Bank. These classification models were developed through five input variables such as age, systolic blood pressure, heart rate, respiration rate and consciousness score.

### Data source

We used information from the National Trauma Data Bank (NTDB) [22], which is the largest traumatic injury dataset; this was assembled by the American College of Surgeons. The NTDB contains over 3 million records, which were voluntarily submitted by over 900 U.S. trauma centers. Among the NTDB, we selected the National Sample Project (NSP) dataset, which was collected separately by 100 trauma centers. The initial dataset was composed of NSP data collected between 2007 and 2013. From this dataset, we extracted incident cases where patients were transported with emergency medical services; patients must also have been over 18 years of age. The records of the NSP dataset were classified by the diagnostic code ICD-9 and can be grouped into specific injury mechanisms. Among more than 25 different injury mechanisms, we selected cases with blunt and penetrating injuries, which are analogous to the traumatic injuries suffered during MCI situations. Each incident case contains data from either the emergency medical service (EMS) or the emergency department (ED).

Among the selected dataset, there are some cases annotated with missing code values. Missing codes means that the variables are not applicable to the case or not recorded during hospitalization. There are three methods that can be used to treat missing data: discarding data, parameter estimation, and data imputation [23]. Among these methods, we applied the complete case analysis [24], which discards samples that contain missing variables. This method is suitable when the missing data are completely random [23] and do not depend on other known values or distributions. Therefore, we excluded cases that contain missing variables from this study.

### Selection of input variable

In this study, five variables were used to develop the casualty classification model. We selected biological features that can be measured with wearable devices. Based on previous studies, a patient’s age is known to have a negative correlation with their survival probability [25]. Therefore, we also included age as a basic characteristic of patients. The systolic blood pressure (SBP), heart rate (HR), and respiration rate (RR) are also included as input variables based on vital signs. In addition to these variables, we proposed a new coma scale, referred to as the simplified consciousness score (SCS), which adjusts GCS when a patient is equipped with a wearable device. The proposed SCS is made up of two types of responses: verbal and motor. Each response is determined based on a binary decision, as shown in Table 1. The scoring method of SCS was based on the GCS classification criteria and can be discriminated by a wearable device. Verbal and motor responses were determined based on a consciousness-based response rather than on simple sound response. Thus, the verbal response was determined when any words were detected, and the motor response was determined by considering the response to local stimulation only.

**Table 1:** Score table of Glasgow coma scale (GCS) and simplified consciousness score (SCS).

Criteria		GCS score	SCS score
**Eye opening**	Spontaneous	4	-
	To speech	3	
	To pain	2	
	None	1	
**Verbal response**	Oriented	5	2
	Confused conversation	4	
	Inappropriate word	3	
	Incomprehensible sounds	2	1
	None	1	
**Motor response**	Obeys commands	6	2
	Localizes pain	5	
	Normal flexion (withdrawal)	4	1
	Abnormal flexion (decorticate)	3	
	Extension (decerebrate)	2	
	None	1	
**Total score**		3–15	2–4

### Outcome

The primary outcome was the recorded survival or death after discharge into the emergency department. This record was documented in the ED chart of the NSP dataset. To delimit the cause of death clearly, cases of death were confined to patients who died in the emergency department before being discharged to other places, like an operating room or a patient’s home.

### Machine learning algorithms

To develop the classification model for survival prediction, we selected three methods. Logistic regression [19] is a basic machine learning method for binary decision making in the medical field. It is a linear model that estimates the logarithmic probability of a dependent variable (target class) as a linear combination of independent variables (input features). The random forest method [20] is a kind of ensemble learning algorithm that fuses several models as base classifiers to produce the output. It has the advantage of reducing the variability of models. The random forest method uses decision trees as the base learner and bagging as the ensemble method. The bagging method creates diverse models by randomly partitioning the training data (i.e., randomly sampling the training examples or randomly selecting a subset of features) and produces outputs by combining the model with major voting. Neural network [21] is a machine learning algorithm that emulates the synaptic structure of the brain. It consists of three types of layers: the input layer, hidden layers, and the output layer. The output of each layer node (except for the input layer) consists as a weighted linear combination of the output of the previous layer nodes that were transformed by a nonlinear function, such as a rectified linear unit (RELU) [26]. This characteristic of nonlinearity enables the neural network to learn complex relationships between input variables, which can increase performance as a data-driven machine learning model [27, 28].

When it comes to developing machine learning algorithms, the total dataset was split into a training set and a test set. The training set is used for deriving the survivability prediction algorithms and the test set is used for evaluating the derived algorithms. This process guarantees the generalized performance of the trained model for new incoming data that has never been seen before. To evaluate the generalized performance, we used a stratified 10-fold cross-validation test. This randomly divides all the data into 10 partitions (folds), then trains a model with nine of the partitions and tests the model with the remaining fold. This process is repeated 10 times by changing the folds for training and testing, evaluating the performance of the models at the end. These processes guarantee the generalized performance of a model by avoiding the risk of overfitting to the samples. However, the dataset has a small number of samples for the death class (less than 3.89%), so random sampling has the risk of creating a fold without samples from the death class. Therefore, we applied stratified sampling [29], which guarantees that each fold maintains the same ratio between classes as the total population.

### Classification model for survival prediction

First, to develop the survival prediction algorithm, we compared the influence of the input variables via the logistic regression, which is the basic machine learning algorithm that is used in RTS and TRISS [8,9]. We developed and compared the discriminative power of each logistic regression model with different input variable compositions using the vital signs, vital signs with GCS, and vital signs with SCS. Second, we compared the performance of the machine learning algorithms. Thus, we trained the survivability prediction model via the logistic regression, random forest, and deep neural network methods. Among these machine learning algorithms, the random forest and neural network algorithms are sensitive to the balance of classes in the training data. Therefore, we applied the weighted cost for learning of each algorithm. When it comes to training machine learning, certain hyperparameters can be tuned empirically to improve the performance of algorithms. In the case of the random forest algorithm, we tuned the size of the leaf, the maximum number of splits for branches, the number of input variables to the sample, and the number of trees. The deep neural network structure consists of two hidden layers, which have 128 nodes at each. We applied the dropout [30] layer in the network for regularization. We set the batch size as 64 and performed training for 60 epochs. We also performed learning rate scheduling to avoid overfitting. We set the learning rate as 0.0001 at first, and then decreased the rate by 1/10 at 20 and 40 epochs. During these training procedures, we stopped the training process when the validation performance reached its peak. We used the Adam Optimizer [31] for optimization of the network. Finally, we developed a survivability prediction algorithm with the neural network, which showed the most discriminative power, and compared the influence of SCS and GCS. The development of these survivability prediction algorithms was conducted with MATLAB Release 2017b (MathWorks, Inc., Natick, Massachusetts, United States) software, and the neural network process was conducted with the tensorflow [32] library in Python.

### Statistical analysis

The characteristics of the study population were described for each dataset, including the mean and standard deviation for each variable. The performance of machine learning survivability prediction algorithms, developed from the training dataset, was evaluated using the test data by calculating the area under the receiver operating characteristics curve (AUC) [33]. The AUC values of models were also statistically compared with the Wilcoxon signed-rank test [34]. Standard errors and 95% confidence intervals were also estimated for the AUC values using a jack-knife procedure [35]. Statistical analyses assessing algorithm performance were performed using GraphPad Prism version 5.00 for Windows (GraphPad Software, San Diego California USA, www.graphpad.com) and MATLAB Release 2017b (MathWorks, Inc., Natick, Massachusetts, United States) software.

## Results

### Data extraction

**Fig 1:**
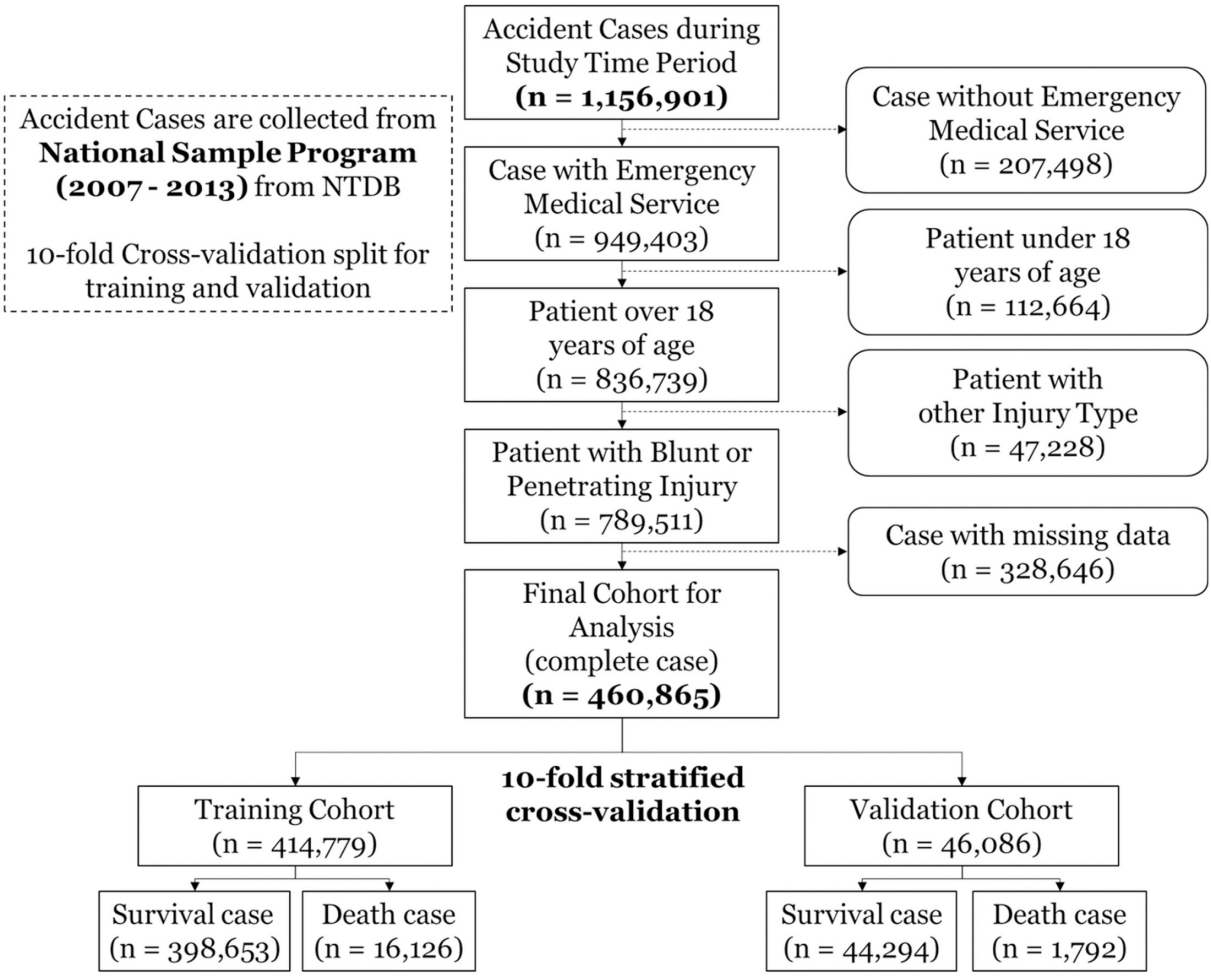
The flowchart for patient data extraction procedure.

A total of 1,156,091 recorded incidents were collected from the NSP dataset for 7 years (from 2007 to 2013), of which 460,865 cases met our criteria (Fig 1). The refined dataset contains 408,316 cases of blunt injury and 52,549 cases of penetrating injury from EMS. In Table 2, the input variables show a significant difference (p < 0.001) between death and survival.

**Table 2:** Characteristics of each input variable from the patient data in the NTDB.

Input Variable	Units	Death	Survival	P-value*
AGE	years (SD)	58.4 (22.7)	48.8 (21.1)	<0.001
SBP	mm HG (SD)	122.9 (53.1)	134.9 (29.8)	<0.001
HR	beat per minute (SD)	86.6 (34.9)	90.7 (20.9)	<0.001
RR	breath per minute (SD)	15.1 (9.8)	18.4 (5.3)	<0.001
SCS	score (SD)	2.9 (1.0)	3.8 (0.5)	<0.001
GCS	score (SD)	8.5 (5.3)	14.0 (2.6)	<0.001

* The p-values were estimated using one-way analysis of variance

### Prediction performance

The performance was evaluated with the AUC value, which represents the discriminative power of the proposed algorithm for survival prediction. We conducted experiments investigating the influence of SCS as the input features for survival prediction. We trained three types of logistic regression models with the same training data, but each model used different input variables. One model was trained with vital signs only, another model was trained with vital signs and the GCS score, and the final model with trained with vital signs and the SCS score. In Table 3, the AUC values of the model that were developed with vital signs and SCS or GCS showed significantly increased performance relative to the models that were developed using only vital signs. The performance was comparable between the models that used SCS and GCS as input variables.

**Table 3:** Performance of the machine learning algorithms for survivability predictions.

Algorithms	Input Variables	Mean AUC	Standard Deviation	95% Confidence Interval*	
				LCL	UCL
RTS (Baseline)	SBP, RR, GCS	0.78	0.007	0.775	0.785
Logistic Regression	Age, SBP, RR, HR	0.71	0.009	0.705	0.718
Logistic Regression	Age, SBP, RR, HR, SCS	0.87	0.005	0.862	0.870
Logistic Regression	Age, SBP, RR, HR, GCS	0.88	0.005	0.872	0.880
Random Forest	Age, SBP, RR, HR, SCS	0.87	0.007	0.862	0.872
**Neural Network**	**Age, SBP, RR, HR, SCS**	**0.89**	**0.005**	**0.882**	**0.890**
Neural Network	Age, SBP, RR, HR, GCS	0.89	0.007	0.885	0.895
TRISS	Age, SBP, RR, GCS, ISS	0.90	0.005	0.901	0.909

* The standard errors were estimated by the jackknife procedure for 10-fold cross-validation results.

The AUC values for each machine learning method and conventional models are also described in Table 4. All the machine learning algorithms showed improved performance relative to RTS, which showed a mean AUC value of 0.78±0.007. The logistic regression and random forest models showed AUC values of 0.87±0.005 and 0.87±0.007, respectively. However, the deep neural network model achieved the best AUC value of 0.89±0.005, which is comparable with the performance of TRISS (0.90±0.005). The performance of TRISS seems to be slightly higher, but TRISS consists of two models that are developed for blunt and penetrating trauma separately. Therefore, the performance of the TRISS model was separately computed with the blunt trauma group and penetrating trauma group.

**Table 4:** Comparison of false positive ratio (FPR) and true positive ratio (TPR) for machine learning algorithms for survivability predictions.

Algorithms	Input Variables	FPR (%)	TPR (%)
RTS (Baseline)	GCS, SBP, RR	33.9	85.8
Logistic Regression	Age, SBP, RR, HR, SCS	21.2	78.8
Random Forest	Age, SBP, RR, HR, SCS	20.8	77.5
**Neural Network**	**Age, SBP, RR, HR, SCS**	**19.7**	**79.7**
Neural Network	Age, SBP, RR, HR, GCS	19.5	80.9
TRISS	Age, SBP, RR, GCS, ISS	18.5	84.0

### Classification analysis

**Fig 2:**
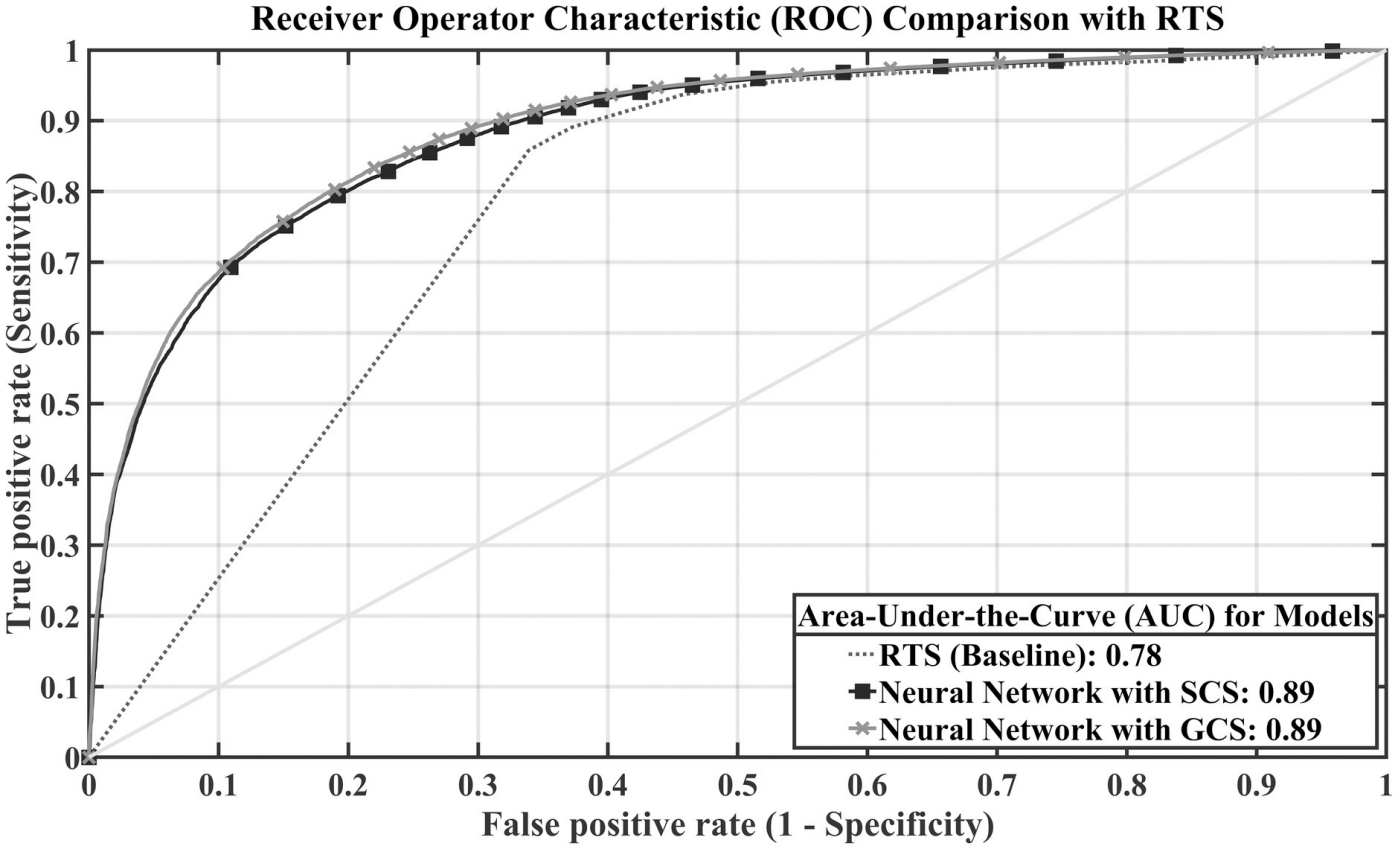
ROC curves with AUC values for the neural networks and RTS. Comparison between the neural network with simplified consciousness score (SCS), the neural network with Glasgow coma scale (GCS) and existing triage model, RTS. Neural networks developed without the injury severity score (ISS) outperformed the revised trauma score (RTS).

We analysed and compared the false positive ratio (FPR) and true positive ratio (TPR) for each machine learning and conventional model. The FPR and TPR values were computed with the optimal operating point in the ROC curve, in which the classifier shows the best performance. In Fig 2, the neural networks developed with GCS and SCS achieve similar ROC curve and same AUC scores and they outperformed the RTS model. The deep neural network model showed an FPR of 19.7% and a TPR of 79.7%, while RTS showed values of 33.9% and 85.8%, respectively. We also compared the predicted survival scores for survivor and death cases for the test dataset with the neural network model. In Table 5, the survival class showed an average predicted survival score of 0.9906 in the total EMS dataset, while the death class showed a value of 0.8661. We also computed the predicted scores for the blunt trauma groups and penetrating trauma groups. The predicted score for the survivor class was 0.9910, while the death class showed a score of 0.8673 in the blunt trauma groups. Similarly, in the penetrating trauma groups, the survivor class score was 0.9877 while the death class scored 0.8603. These predicted survival scores showed a significant difference (P < 0.0001) between survivors and deaths, both overall and for each trauma group.

**Table 5:** Comparison of the predicted survival score for the total dataset and each injury mechanism with the neural network.

	Total Dataset	Blunt Dataset	Penetrating Dataset
**Survivors** (P_mean_ ± SEM)	0.9906 (± 0.00008)	0.9910 (± 0.00008)	0.9877 (± 0.0003)
**Deaths** (P_mean_ ± SEM)	0.8662 (± 0.0015)	0.8673 (± 0.0004)	0.8603 (± 0.0032)

### Ranking of machine learning variables

We evaluated how well each input variable worked in combination as well as how much each variable contributed to the models. For logistic regression, we computed the odds ratio of each input variable from our trained modes. In the case of the random forest method, we estimated the predictor importance by summing the change of the risk due to splits on every variable and dividing the sum by the number of branch nodes in the trained models. For the deep neural network method, we performed sensitivity analysis of the neural network [36,37] and created a benchmark dataset as the input of the neural network in order to measure the relationship between the input and output of trained models. Table 6 shows the results of feature importance analysis for the logistic regression, random forest, and neural network algorithms. In the logistic regression model, SCS is proven to be the most important feature for survival prediction, while SBP is determined to be the second-most important feature. The random forest and neural network methods also showed that SCS is the most important variable.

**Table 6:** Feature ranking of the machine learning algorithms; a lower number indicates a greater importance.

Ranking	Age	SBP	RR	HR	SCS
Logistic Regression	5	2	3	4	**1**
Random Forest	4	2	3	5	**1**
Neural Network	5	3	2	4	**1**
Average Ranking	4.6	2.3	2.6	4.3	**1**

## Discussion

Firefighters who engage in rescue operations related to terrorism, fire, and natural disasters or combatants involved in battlefield operations are exposed to the risk of death. Combatants are at high risk of traumatic injuries and firefighters may be exposed to unforeseen accidents such as secondary collapse during rescue operations in the field. To prepare for these types of trauma situations at remote sites, attempts have been made to classify patients based only on the continuous measurement of their vital signs [38,39]. However, it is not accurate to estimate the survival of trauma patients based only on their vital signs because there are homeostatic mechanisms in the human body that attempt to maintain consistency against internal and external changes. Although a patient’s heart rate and respiration rate are remarkably changed in the case of hypovolemic shock [40,41], these are not enough to accurately evaluate trauma patients. Therefore, it is necessary to develop a casualty classification model with high prediction accuracy while minimizing the time and manpower needed to reach a diagnosis in order to increase the survival rate in mass casualty incidents.

For this purpose, this study first focused on the development of SCS, which adjusts GCS values for remote monitoring. A study by Gill et al., conducted in 2005, showed only a 3% difference between the simplified score (0–3 points) and the GCS score (3–15 points) when predicting the outcome of traumatic brain injuries [11]. Also, GCS, which has high complexity, was reported to have a low interrater reliability [42], and it was confirmed that the interrater reliability of the simplified scale of GCS was highest among various types of consciousness evaluation methods (e.g., GCS, simplified scale, AVPU, ACDU, etc. [12]). Based on the results of these studies, we believe that simplifying GCS enables monitoring through smart or wearable devices. The verbal response can be categorized via smart devices [43]; if a verbal response can be recognized by automatic speech recognition technology via a smart device, it is scored as 2 points (and 1 point otherwise). Additionally, the motor response can be scored based on the response to local pain through a wristband or earphone. To test the motor response, a stimulus can be applied to the wearable device, and the injured patient can directly turn off the stimulus. If the stimulus is turned off, it is scored as 2 points (and 1 point otherwise).

Before developing the machine learning algorithms, we statistically analysed each variable via one-way analysis of variance. As shown in Table 2, the selected input variables were significantly different between the death and the survival for most of the dataset. We also quantified the importance of each feature in determining the survivability of the developed algorithms. Our analysis revealed that the level of consciousness, e.g., the GCS or SCS score, is the most important feature for survivability prediction. Moreover, in Table 3, the logistic regression survivability prediction model with SCS showed slightly inferior performance compared to the logistic regression model with GCS. For the deep neural network algorithm, however, the performance difference between using GCS and SCS as input features was not significant (Table 4). Since the logistic regression handles the input variables linearly, the informative loss of SCS deteriorated the survivability prediction performance. However, the neural network features nonlinear characteristics when learning the complex relationships between input features, which can compensate for the loss of information [27, 28]. Furthermore, when comparing the machine learning algorithms, RTS was basically developed with the logistic regression. The random forest and neural network methods have more discriminative power due to their nonlinear relationship between the input and output. Therefore, the random forest and the neural network algorithms showed improved performance compared to the logistic models. This is also due to the nonlinear characteristic of the neural network, which computes non-linear input-output mapping to express a hyperplane for discrimination [27, 28].

**Fig 3:**
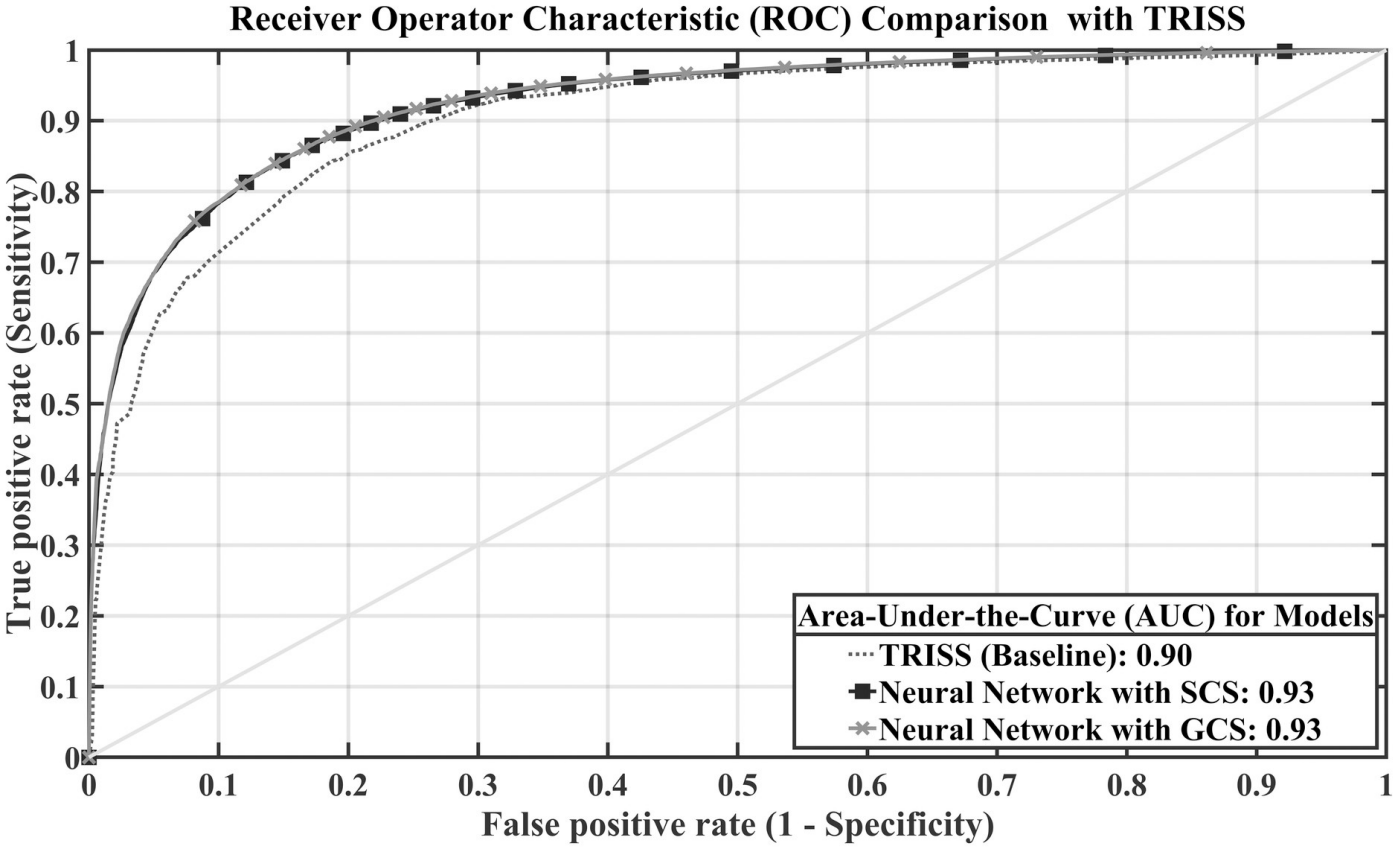
ROC curves with AUC values for the neural network and TRISS. Comparison between the additionally developed neural network models with the injury severity score (ISS) and existing triage model, TRISS. In both model, with SCS and GCS, the neural network developed with the ISS also outperformed the trauma and injury severity score (TRISS).

We proposed the development of a unified remote triage algorithm for blunt and penetrating trauma; however, TRISS requires the ISS score, which is difficult to automate as an input variable. Therefore, we excluded ISS as an input variable in this research. However, to investigate the applicability of machine learning to triage, we also trained and tested the deep neural network model with the same input variables as TRISS. This method achieved an AUC value of 0.93±0.005, which is significantly improved compared to TRISS (Table 7). In Fig 3, the neural networks developed with GCS and SCS achieve similar ROC curve and same AUC scores and they outperformed the TRISS model. Additionally, we also trained and tested our neural network model in the ED dataset. Although the triage situation is different, the deep neural network method showed similar performance with an AUC value of 0.90±0.002. Based on the results of the current study, the machine learning approach for remote triage showed comparable performance with a standard triage model (i.e., TRISS). However, the proposed approach can be automated and is implantable with a wearable device, which will be useful in prehospital environments.

**Table 7:** Performance of the machine learning algorithms for survivability predictions. The standard errors were estimated by the jack-knife procedure.

Algorithms	Input Variables	AUC	Standard Error	95% Confidence Interval	
				LCL	UCL
TRISS	Age, SBP, RR, GCS, ISS	0.90	0.005	0.901	0.909
**Neural Network**	**Age, SBP, RR, HR, SCS, ISS**	**0.93**	**0.005**	**0.921**	**0.929**
Neural Network	Age, SBP, RR, HR, GCS, ISS	0.93	0.005	0.922	0.930

### Limitations

This study used the secondary retrospective dataset from the NTDB and has the limitations about the bias of the dataset which exist in any retrospective study. Most of the dataset is collected from patients who suffer an accident in daily life rather than a disaster. Therefore, the deficiency of samples collected from the patients in a disaster is the limitation of this study. Nonetheless, the dataset contains various injury mechanism that may occur in a disaster site. We expect that our approach is applicable to the triage of patients at the site of a disaster with the practical optimization in the field.

Our machine learning-based survivability prediction algorithm achieved comparable performance to TRISS in terms of its discriminative power, as represented by the AUC value. The predicted score value itself has a biased output toward 1 for survival (0.9906) and death (0.8662), which could be caused by the imbalance of the dataset between death and survival. Therefore, to practically implement our approach in the field, a practitioner who comprehends the output meaning of machine learning should scale the output with the field data to provide a more conclusive probability. Even though the proposed SCS achieved improved survivability prediction performance with this data-driven machine learning approach, the SCS data that are used to develop and evaluate the algorithm are reproduced from GCS values of the NTDB data. Therefore, the SCS values and the developed survivability prediction algorithm should be evaluated with real field data collected in wearable situations. However, the current improvement of wearable device technology indicates that this may be achieved in the near future.

## Conclusions

The goal of this study was to develop a survival classification model that can quickly and automatically triage via wearable devices without medical personnel. For this purpose, we proposed a simplified consciousness score that does not rely on the presence of medical personnel and developed a classification model of survival prediction with high accuracy based on vital signs. In conclusion, we investigated the possibility to triage remotely through wearable devices. The classification model for survival prediction is expected to effectively reduce the time for triage and increase the survival rate of injured patients in prehospital environments during mass casualty incidents.
